# Analysis of Under-Five Mortality Rate in Saudi Arabia: 1973 to 2022

**DOI:** 10.7759/cureus.78048

**Published:** 2025-01-27

**Authors:** Abdullah Al-Nafeesah

**Affiliations:** 1 Department of Pediatrics, College of Medicine, Qassim University, Unaizah, SAU

**Keywords:** newborn, saudi arabia, sex, under-five mortality rate, unicef

## Abstract

Introduction: The under-five mortality rate (U5MR) refers to the probability that a newborn will die before reaching exactly five years of age. The U5MR is expressed per 1,000 live births. Saudi Arabia has made efforts to achieve the sustainable development goals (SDGs), for child mortality by 2030. Although Saudi Arabia has put in place initiatives to achieve the SDGs for child mortality by 2030, data on the success of these initiatives are lacking. The aim of this study was to investigate and compare U5MR trends and patterns in Saudi Arabia by decade and sex over a 50-year period (1973-2022).

Methods: Data on the U5MR in Saudi Arabia during 1973-2022 were extracted from the UNICEF database. The mean mortality rates every 10 years were calculated and then compared over decades between males and females using a chi-square test. A two-sided P-value of < 0.05 was considered statistically significant.

Results: The U5MR was highest in 1973 (149.1 per 1,000 live births; 150.7 males vs. 147.3 females) and lowest in 2022 (6.4 per 1,000 live births; 6.6 males vs. 6.3 females). The statistical analysis revealed a significant decline in the mean U5MR during 1973-2022 (P < 0.01), with no difference between male and female U5MR in any of the five decades.

Conclusions: The analysis of U5MR trends over five decades in Saudi Arabia (1973-2022) revealed a remarkable decline in the rate among both males and females, with a U5MR of 149.1 per 1,000 live births in 1973 and 6.4 per 1,000 live births in 2022. Saudi Arabia has achieved the SDG target 3.2 (ending preventable deaths of newborns and children under five years of age by 2030). Based on an analysis of the UNICEF data and a literature review, recommendations are proposed to achieve even greater reductions in the U5MR, especially in the neonatal and infant stages.

## Introduction

The under-five mortality rate (U5MR) refers to the probability that a newborn would die before reaching exactly five years of age [1]. The U5MR is expressed per 1,000 live births [1]. According to the United Nations Children’s Fund (UNICEF), in 2022, 4.9 million children under the age of five years died worldwide, which translates to 13,400 deaths per day [2]. Globally, infectious diseases, including pneumonia, diarrhea, and malaria remain leading the causes of death in children under the age of five, along with preterm birth and intrapartum-related complications [1,3]. The U5MR is an indicator of child health, and a population's overall development and well-being [1]. Many studies have reported nutritional and healthcare factors that have a significant impact on under-five morbidity and mortality [4-6]. These factors include low birth weight (LBW), delayed initiation of breastfeeding, neonatal complications, maternal complications, and lack of access to healthcare services [4-6]. U5MR serves as a crucial indicator of child health and the effectiveness of the healthcare systems of a country. In Saudi Arabia, significant strides have been made to reduce U5MR over the past few decades, reflecting improvements in healthcare access and quality [7-9]. As of 2020, the U5MR in Saudi Arabia was reported at approximately 7.0 deaths per 1,000 live births, a considerable decline from 44.3 deaths per 1,000 live births in 1990 [9]. This decrease is indicative of successful health interventions, including enhanced maternal and child healthcare services, widespread immunization programs, and improved socioeconomic conditions [7,8,10].

Although in Saudi Arabia, significant progress has been made in reducing U5MR over the past few decades [7-9], the differences between genders were not explored. Some studies indicate that females face a higher risk of childhood mortality than males, particularly in nations where gender bias in healthcare services is documented [11,12]. Conversely, other studies, such as those conducted in India, report that males have a higher risk of mortality [13]. Addressing the gender-specific factors influencing U5MR is essential for developing targeted interventions aimed at further reducing child mortality in Saudi Arabia. In addition, understanding these dynamics will provide insights for healthcare policymakers and practitioners to implement effective strategies that ensure equitable health outcomes for all children, regardless of gender. Although previous studies have reported on under-five mortality in various Gulf countries [10,14], including Saudi Arabia [15], at different times, none of these have analyzed trends in the U5MR rate over decades. UNICEF data on U5MRs provide a reliable source of information on trends at the country level. Saudi Arabia is the largest Gulf country, consisting of 13 regions. Riyadh, the capital of Saudi Arabia, has a population of 31.5 million, of whom the majority live in urban areas, with only 17% living in rural areas [16]. According to the World Health Organization (WHO), the under-five population accounted for 3.2 million of the total population (i.e., one-third of the total population) [16]. In addition, in Saudi Arabia, all births are registered [17], which increases the reliability of UNICEF data regarding the health of under-five children. The aim of this study was to investigate U5MR trends and patterns in Saudi Arabia during a 50-year period (1973-2022) and to compare differences between the mortality rates of males and females during this period. Based on an analysis of the UNICEF data and a literature review, recommendations are proposed aimed at further improving under-five health in Saudi Arabia and other Gulf countries.

## Materials and methods

UNICEF gathers data on U5MR in various countries, including Saudi Arabia, using various methodologies to ensure thorough and precise assessments. The main methods employed by UNICEF to gather data regarding U5MR include but are not limited to [9,18,19].

National surveys

UNICEF collaborates with the Saudi government to conduct health surveys, such as the multiple indicator cluster surveys (MICS) and demographic and health surveys (DHS). These surveys gather data on various health indicators, including child mortality, through structured household interviews.

Vital registration systems

Data were collected from the national civil registration and vital statistics systems, which track births and deaths. This system provides essential information for calculating mortality rates and understanding demographic changes over time.

Administrative data

UNICEF utilizes data from the Saudi Ministry of Health and other governmental agencies that maintain records on health indicators, including child health and mortality statistics.

Collaboration with local partners

By partnering with local non-governmental organizations and community organizations, UNICEF collects qualitative and quantitative data that provide insights into the challenges and barriers affecting child health in different regions of Saudi Arabia. These combined efforts enable UNICEF to provide reliable estimates of U5MRs in Saudi Arabia and support the development of effective policies and programs aimed at improving child health outcomes. 

In this study, the target population was the under-five children born during the period from 1973 to 2022 in Saudi Arabia. The primary outcome of this study was the U5MR among both genders during the period from 1973 to 2022. U5MR data for the period 1973-2022 were obtained from the UNICEF database [9]. In line with this study, previous studies have used the World Bank database for a similar purpose [20]. The data were downloaded in Excel file (Microsoft Corporation, Washington, DC) format. Open Epi (Centers for Disease Control and Prevention, Atlanta, GA) was utilized to calculate the means and the differences in the U5MR between the means. The downloaded data were checked for completeness for both the U5MR number in each year from 1973 to 2022 as well as for both genders, male and female, and found no missing data regarding the U5MR number and gender. The mean mortality rates every 10 years were calculated starting from the year 1973 to 2022 and compared between decades and between males and females using a chi-square test. A two-sided P-value of < 0.05 was considered statistically significant.

Ethics approval

Ethical approval was not required for this study, as the data on under-five mortality in Saudi Arabia are available from the UNICEF website and can be used without permission. Therefore, for such kinds of study, no need to seek ethical approval from the institutional board.

## Results

**Figure 1:**
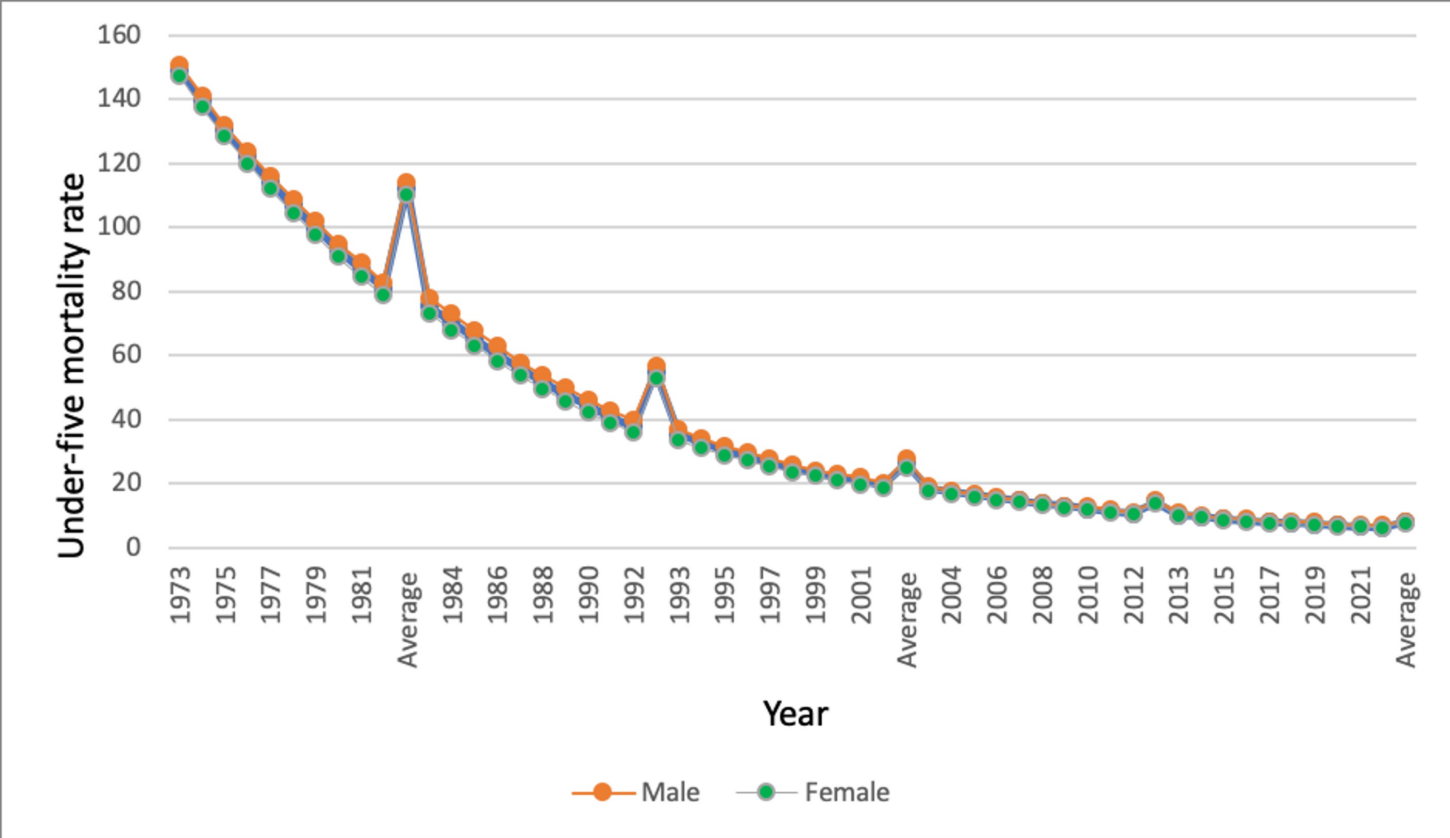
Under-five mortality (U5MR) in Saudi Arabia during 1973-2022. The source of this figure data is the UNICEF [9]. The average values placed along the X-axis on the graph represent the average of under-five child deaths per 1,000 live births over a decade.

The U5MR was highest in 1973 (149.1 per 1,000 live births; 150.7 for males vs. 147.3 for females) and lowest in 2022 (6.4 per 1,000 live births; 6.6 for males vs. 6.3 for females). Figure 1 shows the overall gradual decline in the U5MR from the first to the fifth decade among males and females. The U5MR was highest in the first decade (1973-1982) at 112.33 per 1,000 live birth (114.0 for males vs. 110.2 for females), and declined in subsequent decades, with 55.09 (57.0 for males vs. 52.97 for females), 26.45 (28.0 for males vs. 26.23 for females), 14.41 (15.0 for males vs. 13.95 for females), and 8.07 (8.0 for males vs. 7.85 for females) deaths per 1,000 live births in the second (1983-1992), third (1993-2002), fourth (2003-2012, and fifth (2013-2022) decade, respectively.

Table 1 compares the U5MR over five decades (1973-2022) for males and females [9]. As shown in the table, there was a significant decline in the mean U5MR among both males and females over the five decades (P < 0.01), with no difference between the U5MR of males and females in each decade in a chi-square test (P > 0.05).

**Table 1:** Under-five mortality rate (U5MR) of males and females in Saudi Arabia over five decades (1973–2022). The source of this table data is the UNICEF.

Decade	Total	Males	Females	P-value of the difference between genders
1973‒1982	55.09	57.07	52.97	0.687
1983‒1992	26.45	27.57	25.23	0.744
1993‒2002	19.6	20.3	18.8	0.808
2003‒2012	10.8	11.1	10.5	0.896
2013‒2022	6.4	6.6	6.3	0.933
P-value of the difference across decades	<0.001	<0.001	<0.001	

## Discussion

The main finding of this study is the remarkable decline in the U5MR over the last fifty years among both males and females. This finding agrees with the regional contexts in Arab Gulf countries [10] and Saudi Arabia [20,21]. Al-Mazrou et al. revealed a continuous and marked decline in infant and child mortality during 1994-2004. In their study, the infant mortality rate (the number of deaths of children under one year of age) declined from 22 per 1,000 live births in 1994 to 17 per 1,000 live births in 2004, and the U5MR declined from 34 to 22 per 1,000 live births during the same period [21]. The decline in the U5MR found in our study is in accordance with the global trend in U5MRs, which have fallen by more than 50% since 2000. According to UNICEF, this remarkable achievement has been driven mainly by sustained commitment by governments, organizations, local communities, healthcare workers, and families [2]. This finding confirmed the achievement of Saudi Arabia of the proposed Sustainable Development Goal (SDG) target for child mortality, which aims to reduce the U5MR to at least 25 deaths per 1,000 live births. Likewise, Shawky reported a decline in the infant mortality rate from 75 per 1,000 live births in 1978 to 23 per 1,000 live births in 1999 (a declining rate of 69.3%) [21,22]. In contrast to a child born in Australia and New Zealand, one born in sub-Saharan Africa is, on average, 18 times more likely to die before reaching the age of five years [2]. Several factors, such as improvements in education, socioeconomic status, and health services, may account for the remarkable decline in the U5MR in Saudi Arabia [20]. In Zambia, Jacobs et al. attributed a decline in the U5MR from 168 to 64 deaths per 1,000 live births between 2001 and 2018 to government policies aimed at combating socioeconomic inequalities, including inequalities relating to education and urban-rural residence groups [23]. In Saudi Arabia, with the aim of further reducing neonatal, infant, and under-five mortality, more healthcare workers were recruited to ensure high-quality care for mothers and children, especially in the early years. Today, 98.7% of births in Saudi Arabia are attended by healthcare workers [24]. A similar healthcare initiative (i.e., expanding the healthcare workforce) has been successful in Bangladesh [25].

Greater availability of healthcare workers could have contributed to the expansion of vaccine coverage in Saudi Arabia. Therefore, the observed decline in the U5MR in the present study could be due to the greater immunization coverage [7]. For example, the expansion of rotavirus immunization programs globally resulted in a remarkable reduction in under-five mortality [26]. Assuming global use of rotavirus vaccines and coverage equivalent to other co-administered vaccines, Clark et al. concluded that they could prevent 37% of under-five rotavirus-related deaths (1.2% of child mortality) [26]. In addition, the decline in the U5MR in Saudi Arabia could be due to improvements in water, sanitation, and hygiene (WASH) services [10,21,22]. Salam and Al-Khraif attributed the remarkable decline in neonatal, infant, and U5MR since 1950 to a significant budget allocation and investment in health system building, including WASH, nutrition, and lifestyle modifications [10]. Previously, lack of access to adequate WASH services was the main factor reported to be associated with infant mortality in Arab countries, including Saudi Arabia [22]. Recently, Gaffan et al. reported that the risk of under-five mortality was 11% higher for children living in households with poor or no sanitation facilities compared to those with basic sanitation services [27].

In this study, a decline in the U5MR was found in both males and females. Some studies reported that females had a higher risk of childhood mortality than males, especially in countries with evidence of gender bias in healthcare services [11,12]. However, in India, Pal et al. found that the risk of under-five mortality was higher among males than females [13]. The decline in the U5MR among both males and females in Saudi Arabia may point to equality in the provision of healthcare to pregnant women and their children, regardless of the sex of the child.

Aiming to empower women, North African and Middle Eastern countries, including Saudi Arabia, have focused on access to education over the past few decades [24]. As a result, there have been dramatic improvements in education for all, with several encouraging trends in the primary and secondary education of females [28]. Over the years, Saudi Arabia has taken essential steps in girls’ education, which might have contributed to the decline in U5MR found in the present study. In a recent survey, Hassan reported that maternal education had a positive impact on reducing under-five morbidity and mortality in sub-Saharan Africa by empowering mothers to make decisions regarding their health [29]. On the other hand, lack of maternal education has a negative impact on child morbidity and mortality in many countries, including Saudi Arabia [21,30-32]. In 1998, the male-to-female literacy ratio in Saudi Arabia was 80:60 (1.4) [22]. Since then, the Saudi government has put in place several initiatives aimed at improving the education of females [8]. These include increasing access to education among girls and reducing the gender gap at higher educational levels [8]. A high maternal education level could contribute to a reduction in the U5MR in several ways. For example, it could lead to marriage at an older age, higher earning potential, and changes in residence preferences from rural to urban [21,30]. Ahinkorah reported that children of mothers in sub-Saharan Africa whose first child was born when they were younger than 20 years were 11% more likely to die before the age of five years compared with their counterparts whose mothers’ first childbirth occurred at age ≥20 years [33].

According to the WHO, common causes of under-five mortality in Saudi Arabia are prematurity (22%), congenital anomalies (14.2%), injuries (9.6%), and birth asphyxia/trauma (7.5%) [16]. Although under-five mortality is not categorized by age in the UNICEF database, the WHO has estimated that neonatal deaths (i.e., mortality within the first 28 days) account for 54% of all under-five mortality [34]. In Saudi Arabia, Al-Abbas and Ahmed reported minimal changes in the infant mortality rate between 2003 and 2014 [12]. In the same study, in 2015, the U5MR was 14.5 per 1,000, the infant mortality rate was 12.5 per 1,000 live births, the neonatal mortality rate was 7.9 per 1,000 live births, and the maternal mortality rate was 12 per 100,000 live births. These figures indicate that additional efforts are needed to improve child health, especially in the early stages (neonatal and infant). Such improvement cannot be achieved without more focus on maternal health, specifically the first 1,000 days of life (i.e., nine months of pregnancy and the first two years of a child’s life) [35].

The findings of this study have several implications for maternal and child health in Saudi Arabia. Our analysis of the trend in U5MR in Saudi Arabia revealed a remarkable decline during the study period. This success can encourage policymakers, healthcare workers, and the local communities to continue their efforts to further improve infants’ and children’s health. The collective efforts of the government and the local communities have prevented millions of child deaths since 1973. However, a further decline in the U5MR can be achieved, and it is possible, especially for preventable child deaths. Sharrow et al. predicted 48.1 million under-five deaths worldwide, especially during the neonatal period, between 2020 and 2030, if no further preventative actions were taken [3]. As shown by a review of the literature, there is little published research on the U5MR in Saudi Arabia, despite the remarkable progress that has been made in reducing the rate in recent years. Neonatal deaths account for more than half (54%) of under-five mortality worldwide [16]. There has been little change in the infant mortality rate in Saudi Arabia [20]. Thus, more research is needed to explore the social determinants of under-five mortality, especially those related to maternal health. To reduce the U5MR further in Saudi Arabia, cost-effective interventions are needed that target subpopulations of children at higher risk, especially neonates and infants. Such interventions should be coordinated with United Nations (UN) agencies like UNICEF.

The present study has some strengths. Reporting that Saudi Arabia has already achieved SDG Target 3.2 by reducing its U5MR to 6.4 per 1,000 live births by 2022, which is significantly lower than the target of 25 per 1,000 live births. This achievement reflects the country's successful implementation of healthcare interventions, immunization programs, and improvements in socioeconomic conditions over the past five decades [7,8,10,21,22].

The present study has some limitations that need to be addressed in future research. First, the study relied on secondary data on the U5MR and did not include data on the child’s age, place of residence (urban/rural), or cause of death. For example, Al-Mazrou et al. observed regional variations in infant and child mortality in rural areas and the southern region of Saudi Arabia, which might reflect differences in accessibility to maternal healthcare services and socioeconomic factors [21]. Al-Abbas and Ahmed’s recommendations emphasized this observation: improving accessibility to healthcare services in rural areas and improving maternal education in Saudi Arabia [20]. In India, there is a rural-urban gap in under-five mortality, which may indicate that social and health policy changes are needed to reach rural children from poor families and uneducated mothers [33]. However, residency is unimportant when the child belongs to a high socioeconomic class and has an educated mother [36]. Second, the present study focused on under-five mortality rather than morbidity, a key factor in mortality. These limitations can be overcome by synchronizing efforts to be more precise in future interventions and save budgets. Appropriate coordination between the Saudi Arabian government and UN agencies, such as UNICEF, will be fruitful (i.e., adding the missing data to the UNICEF tools to synchronize efforts).

## Conclusions

The analysis of U5MR trends over five decades in Saudi Arabia (1973-2022) revealed a remarkable decline in the rate among both males and females, with a U5MR of 149.1 per 1,000 live births in 1973 and 6.4 per 1,000 live births in 2022. Saudi Arabia had achieved the SDG target 3.2 related to under-five mortality. Based on an analysis of the UNICEF data and a literature review, recommendations are proposed to achieve further reductions in the U5MR, especially in the neonatal and infant stages.
